# Antimicrobial-resistant enterobacteria in surface waters with fecal contamination from urban and rural communities

**DOI:** 10.1590/0037-8682-0724-2020

**Published:** 2021-03-08

**Authors:** Vanessa Tibolla Moretto, Soraia Machado Cordeiro, Patricia Salcedo Bartley, Luciano Kalabric Silva, Rafael Ponce-Terashima, Mitermayer Galvão Reis, Ronald Edward Blanton, Lúcio Macedo Barbosa

**Affiliations:** 1 Fundação Oswaldo Cruz, Instituto Gonçalo Moniz, Laboratório de Patologia e Biologia Celular, Salvador, BA, Brasil.; 2 Universidade Federal da Bahia, Faculdade de Farmácia, Salvador, BA, Brasil.; 3 Respiratory institute, Cleveland Clinic, Infectious Diseases Department, Cleveland, Ohio, USA.; 4 Mercer University School of Medicine, Department of Medicine, Division of Infectious Diseases, Macon, Georgia, USA.; 5 Universidade Federal da Bahia, Faculdade de Medicina, Salvador, BA, Brasil.; 6 Yale University, School of Public Health, Department of Epidemiology of Microbial Diseases, New Haven, Connecticut, USA.; 7 Tulane University School of Public Health and Tropical Medicine, Department of Tropical Medicine, New Orleans, LA 70112, USA.; 8 Escola Bahiana de Medicina e Saúde Pública, Salvador, BA, Brasil.

**Keywords:** Fecal contamination, Surface water, Enterobacteria, Antimicrobial resistance, ESBL-producing, Carbapenemase-producing

## Abstract

**INTRODUCTION:**

Inadequate wastewater treatment and fecal contamination have a strong environmental impact on antimicrobial resistance (AMR). This study evaluated the profile of AMR enterobacteria and fecal contamination from four surface waters: Jiquiriça-Brejões River and Cabrito, Tororó, and Abaeté Lagoons.

**METHODS:**

We analyzed AMR β-lactamase genes using the polymerase chain reaction method and fecal contamination using Coliscan^®^.

**RESULTS:**

We found high levels of fecal contamination, β-lactamase producers, and AMR genes (bla_OXA-48_, bla_SPM_, and bla_VIM_) in all waterbodies.

**CONCLUSIONS:**

Poor sanitation evidenced by fecal contamination and human activities around these surface waters contributed to the distribution and increase in AMR enterobacteria.

Aqueous environments are ideal for the selection and dissemination of antibiotic-resistant bacteria^1^ ^,^ ^2^. In such environments, bacteria can thrive, be transported over large areas, and, importantly, be exposed to other bacteria, bacteriophages, animals, and humans. Antimicrobial resistance (AMR) occurs naturally, and the historical increase in the use of antimicrobials, both in the area of medicine and livestock rearing, has accelerated this process^3^. Until 2009, a lack of regulation on antibiotics sales in Brazil was associated with the ease in obtaining and using these medications^4^. Carbapenem-resistant and third-generation cephalosporin-resistant Enterobacteriaceae have been classified as priority pathogens according to the World Health Organization (WHO)^5^. These multidrug-resistant bacteria are the main contributors to human morbidity and mortality owing to their high transmissibility from person to person, from animals to humans, and to the environment. Therefore, AMR has become a major threat to human health^6^. In rural environments, AMR occurs because of the use of antibiotics for agricultural purposes and occurs in livestock through contamination of the soil and adjacent rivers^7^. In urban areas, high population density and inadequate basic sanitation have altered the microbiologic balance of aquatic environments, making them reservoirs of AMR bacteria and a potential source of community infections^8^. Here, we evaluated the fecal contamination of surface waters in rural and urban areas and determined the AMR of isolated Enterobacteriaceae. 

## Study sites

The rural river Jiquiriça-Brejões (JB) is located in Jenipapo, municipality of Ubaíra, Bahia, Brazil. The urban water sources, located in three areas of the capital, Salvador, in Bahia, Brazil are as follows: Cabrito Lagoon (CL), Tororó Lagoon (TL), and Abaeté Lagoon (AL). We selected these four sites for water collection according to the following criteria: proximity to dwellings, recreation sites, presence of non-municipal system sewage pipes, and potential for direct contact of the population with the water source.

JB: This river flows through the Jenipapo area, with approximately 650 inhabitants. Jenipapo is located in Bahia’s central-south region, approximately 270 km from Salvador. The main economic activities are agriculture, livestock, and local commerce. 

CL: This small, shallow lake is located in the neighborhood of Alto do Cabrito, a tributary of the Cobre river. According to the Brazilian Institute of Geography and Statistics (IBGE), this neighborhood has approximately 4,472 inhabitants and is one of the oldest neighborhoods in the city of Salvador (population of 3 million, 2013). 

TL: This lagoon is located in the neighborhood of Tororó. People actively use the area around the lagoon for exercising, boating, fishing, and other recreational activities. 

AL: This lagoon is located in Abaeté Metropolitan Park, an environmentally protected area in the neighborhood of Itapuã*.* Locals frequently use it for recreational purposes, including bathing, jogging, and fishing. 

## Water sampling

We collected water samples (n=48) every 3 months, from October 2016 to August 2017. At each time point, 400 mL of water was collected in sterile glass vials at a depth of approximately 30 cm below the surface. The vials were transported in thermal boxes with dry ice until microbiological analysis. 

## Fecal contamination

We identified coliforms using the Coliscan Easygel^®^ kit (Microbiology Laboratories, Goshen, IN, USA), following the manufacturer’s instructions. The count limit established in this study was 1,000 CFU/mL. According to the standards of bathing conditions determined by the Brazilian Federal Environmental Council (CONAMA; Ordinance No. 274/00), waterbodies exceeding 25 CFU/mL total coliforms and 20 CFU/mL total *Escherichia coli* are considered unsuitable for drinking and recreational use.

## Microbiological analyses

We plated 100 μL of the collected water on MacConkey Agar (Merck, Darmstadt, Germany) containing appropriate antibiotics. For carbapenem-resistance screening, 1 μg/mL meropenem (ABL®, Cosmópolis, São Paulo, Brazil) was added, while for cephalosporin-resistance screening, 2 μg/mL cefotaxime (Sigma-Aldrich, USA) was dissolved in the medium (modified from Montezzi et al^9^). Serial dilutions (1:1, 1:10, and 1:100) of the samples were prepared and incubated for 24 h at 36 ± 2 ºC. Colonies with morphological characteristics suggestive of Enterobacteriaceae were inoculated on triple sugar iron (TSI) agar (Neogen, Lansing, Michigan, USA) to determine the fermentation ability. All glucose-fermenting bacteria isolated on TSI were re-isolated on tryptic soy agar (TSA) (Neogen, Lansing, MI, USA) and routed for identification.

Matrix-assisted laser desorption ionization-time of flight (MALDI-TOF) (VITEK-MS^®^, Biomérieux, France) was employed to identify bacterial species. The AMR susceptibility profile was determined using the VITEK-2^®^ automated system for Enterobacteriaceae (Biomérieux, France). Subsequently, bacterial isolates were stored in the tryptic soy broth medium supplemented with glycerol (20%) at −80 °C for future analysis. 

## Detection of AMR encoding genes

*Enterobacter cloacae*, *E. coli*, and *Klebsiella pneumoniae* isolates were analyzed for AMR. Frozen isolates were re-cultured on TSA for 18-24 h at 36 ± 1 °C, and colonies (±5) were resuspended in 100 μL of sterile distilled water for DNA extraction. Each isolate was incubated at 95 °C for 5 min and then centrifuged at 12,000 rpm for 2 min. The supernatant was transferred to another cryotube and stored at −20 °C until use.

The β-lactamase AMR identification (bla_CTX-M_, bla_SHV_, bla_TEM_, bla_KPC_, bla_VIM_, bla_NDM_, bla_SPM_, and bla_OXA-48_) was performed using the conventional polymerase chain reaction (PCR) method using TopTaq Master Mix® (Qiagen, USA), in accordance with several different protocols. A standard annealing temperature was used for all primers. Cycling conditions were as follows: 96 °C for 1 min, 62 °C for 1 min, an extension step at 72 °C for 1 min, repeated for 35 cycles. Each reaction was adjusted to a final volume of 25 μL, containing 3 μL of water (Qiagen, USA), 12.5 μL of TopTaq Master Mix® (2×) (Qiagen, USA), 2 μL of each primer, 2.5 μL of CoralLoad® (10×) (Qiagen, USA), and 5 μL of DNA. Amplified products were visualized on a 2% agarose gel (Invitrogen, Carlsbad, CA, USA), using 8 μL SYBR® Safe (Invitrogen, Carlsbad, CA, USA) per sample.

Positive controls used in the present study included *E. coli* 300 (*TEM*), *E. coli* 455 (*CTX-M*), *Pseudomonas aeruginosa* (*SPM-1*), *P. fluorescens* CCBH 11805 (*VIM*), *K. pneumoniae* ATCC 7000603 (*SHV)*, *Raoultella ornithinlytica* (*OXA-48*), *E. cloacae* CCB 410882 (*NDM*), *and K. pneumoniae* kp13 (*KPC*).

## Statistical analysis

The data were tabulated using Microsoft Excel (Microsoft Corporation, USA) and analyzed with EpiInfo™ software (Centers for Disease Control, USA) to summarize descriptive statistics, such as frequency distributions, means, and standard deviations. The median count of coliforms and *E. coli* isolates were compared using the nonparametric Kruskal-Wallis test, followed by Dunn’s multiple comparison post hoc test using GraphPad Prism™ 9.0.0 (GraphPad Software, USA). P values < 0.05 were considered significant.

The average total coliform (TC) count for JB was 367.0 CFU/mL.At the urban sites of CL, TL, and AL, the average TC counts were 736.0, 149.4, and 154.3 CFU/mL, respectively. The average *E. coli* count in JB was 164.2 CFU/mL, while that in CL, TL, and AL were 405.7, 4.8, and 9.0 CFU/mL, respectively (Table 1). We did not observe a significant difference between the rural and urban areas; however, among the urban sites, there was a significant difference between CL, AL, and TL. AL and TL are near tourist areas and undergo water treatment and local maintenance, though it is inefficient to make the water usable to humans. In contrast, CL is located in a poor neighborhood in a region lacking maintenance or water treatment.

**TABLE 1:** Total coliform (TC) and *Escherichia coli* counts (in CFU/mL) per site.

	Sample 1		Sample 2		Sample 3		Sample 4		Average	
	TC	*E. coli*	TC	*E. coli*	TC	*E. coli*	TC	*E. coli*	TC	*E. coli*
**JB**	186.7	138.0	383.0	127.0	876.0	385.3	22.3	6.3	367	164.2
**TL**	239.4	7.7	63.6	1.0	239.7	4.7	54.0	5.7	149.2	4.8
**CL**	829.3	544.0	1054.0	591.3	669.4	395.7	391.7	91.7	736.1	405.8
**AL**	258.7	6.0	151.7	23.7	107.0	5.3	99.7	5.0	154.3	10.0
**Total of average**	378.5	173.9	413.1	185.8	473.0	197.8	142.0	27.1	351.7	146.2

**JB:** Jiquiriça-Brejões; **CL:** Cabrito Lagoon; **TL:** Tororó Lagoon; **AL:** Abaeté Lagoon. According to CONAMA (ordinance n^o^ 274/00), waterbodies that exceed 25 CFU/mL total coliforms and 20 CFU/mL total *Escherichia coli* are considered unsuitable for use.

We identified 19 different Enterobacteriaceae species from 196 isolates randomly selected for resistance screening. Overall, the most prevalent species at all sites was *E. cloacae* (33%; 65 isolates), followed by *K. pneumoniae* (22%; 44 isolates) and *E. coli* (16%; 31 isolates). We selected all 140 isolates of *E. cloacae*, *E. coli*, and *K. pneumoniae* for AMR susceptibility testing. All *E. coli* (n=5) and *K. pneumoniae* (n=4) isolates from JB were susceptible to all the evaluated antibiotics. For isolates from CL, we found that 10% of *E. coli* (2/21) and 7% of *K. pneumoniae* (1/13) isolates were producers of extended-spectrum beta-lactamase (ESBL)*.* Additionally, 7% of *K. pneumoniae* (1/13) were resistant to carbapenem (ertapenem, imipenem, and meropenem) and piperacillin/tazobac . For isolates from TL, 65% of *E. cloacae* (17/26) isolates were resistant to at least one of the tested carbapenems and were positive for carbapenemase production. For isolates from AL, 22% of *E. cloacae* isolates (2/9) were resistant to gentamicin while 33% of *E. coli* isolates (1/3) were resistant to ampicillin/sulbactam (Table 2).

**TABLE 2:** Antimicrobial susceptibility profile of *E. cloacae, E. coli* and *K. pneumoniae* isolates per site.

Antimicrobial susceptibility	JB		CL		TL		AL		Total	
	N	%	N	%	N	%	N	%	N	%
***Enterobacter cloacae* (total)**	**17**		**13**		**26**		**9**		**65**	
Resistant (R/I)										
SAM	16	94	13	100	9	34	5	56	34	52
SAM, IPM, HODGE +	-	-	-	-	2	8	-	-	2	3
SAM, MEM, HODGE +	-	-	-	-	2	8	-	-	2	3
SAM, ETP, HODGE +	-	-	-	-	13	50	-	-	13	20
GEN	-	-	-	-	-	-	2	22	2	3
Susceptible	1	6	-	-	-	-	2	22	3	5
***Escherichia coli* (total)**	**5**		**21**		**2**		**3**		**31**	
Resistant (R/I)										
SAM	-	-	-	-	-	-	1	33	1	3
SAM, FEP, CXM, CXA, CAZ, CRO, ESBL+	-	-	2	10	-	-	-	-	2	6
Susceptible	5	100	19	90	2	100	2	67	28	20
***Klebsiella pneumoniae* (total)**	**4**		**13**		**4**		**23**		**44**	
Resistant (R/I)										
SAM, FEP, CXM, CXA, CAZ, CRO, ESBL+	-	-	1	7	-	-	-	-	1	2
TZP, ETP, IPM, MEM	-	-	1	7	-	-	-	-	1	2
Susceptible	4	100	11	86	4	100	23	100	42	96
**Total isolates**	26		47		32		35		140	

**JB:** Jiquiriça-Brejões; **CL:** Cabrito Lagoon; **TL:** Tororó Lagoon; **AL:** Abaeté Lagoon; **SAM:** ampicillin/sulbactam; **AMK:** amikacin; **CAZ:** ceftazidime; **CIP:** ciprofloxacin; **FEP:** cefepime; **CRO:** ceftriaxone; **CXA:** cefuroxime/axetil; **CTX:** cefotaxime; CXM: cefuroxime; **ETP:** ertapenem; **GEN:** gentamicin; **IPM:** imipenem; **I:** indeterminate; **MEM:** meropenem; **TZP:** piperacillin/tazobac; **R:** resistant; **ESBL+:** extended-spectrum beta-lactamase positive.

All isolates of enterobacteria from every site were PCR-positive for at least one of the AMR genes tested in this study. From JB, 11/17 (65%) *E. cloacae*, 4/5 (80%) *E. coli*, and 2/4 (50%) *K. pneumoniae* isolates were PCR-positive for at least one of the tested genes. From CL, 6/13 (46%) *E. cloacae*, 3/21 (14%) *E. coli*, and 3/13 (23%) *K. pneumoniae* isolates were PCR-positive for at least one β-lactam resistance gene. From TL, 19/26 (73%) *E. cloacae*, 4/4 (100%) *K. pneumoniae*, and none of the *E. coli* isolates (n=2) were PCR-positive for the tested genes. From AL, 6/9 (66.7%) *E. cloacae*, 2/3 (66.7%) *E. coli*, and 14/23 (47.8%) *K. pneumoniae* isolates were PCR-positive for at least one of the tested genes (Table 3).

**TABLE 3:** AMR gene profiles of *E. cloacae*, *E. coli,* and *K. pneumoniae* isolates per site.

Resistance genes	JB		CL		TL		AL	
	N	%	N	%	N	%	N	%
***E. cloacae***	**11**		**6**		**19**		**6**	
CTX-M	-	-	1	17	1	5	1	17
NDM	-	-	-	-	1	5	1	17
OXA-48	2	18	1	17	3	16	2	33
SPM-1	2	18	1	17	2	11	1	17
VIM	3	28	1	17	4	21	-	-
CTX-M/VIM	1	9	-	-	-	-	-	-
CTX-M/SPM-1	1	9	-	-	-	-	-	-
CTX-M/OXA-48	-	-	-	-	1	5	-	-
CTX-M/OXA-48/VIM	1	9	-	-	1	5	-	-
OXA-48/VIM	1	9	-	-	4	21	-	-
OXA-48/SPM-1	-	-	-	-	2	11	1	17
VIM/TEM	-	-	1	17	-	-	-	-
VIM/SPM-1	-	-	1	17	-	-	-	-
***E. coli***	**4**		**3**		**4**		**14**	
CTX-M	1	25	1	33	-	-	1	50
OXA-48	1	25	-	-	-	-	-	-
SPM-1	-	-	-	-	-	-	1	50
VIM	2	50	1	33	-	-	-	-
CTX-M/VIM	-	-	1	33	-	-	-	-
***K. pneumoniae***	**2**		**3**		**4**			**14**	
CTX-M	-	-	1	33	1	25	3	21
OXA-48	2	100	2	67	1	25	4	29
SPM-1	-	-	-	-	1	25	3	21
CTX-M/VIM	-	-	-	-	-	-	1	7
CTX-M/SPM-1	-	-	-	-	-	-	1	7
CTX-M/OXA-48	-	-	-	-	-	-	2	14
OXA-48/VIM	-	-	-	-	1	25	-	-

**JB:** Jiquiriça-Brejões; **CL:** Cabrito Lagoon; **TL:** Tororó Lagoon; **AL:** Abaeté Lagoon.

Fecal contamination, especially from human sources, is an important route for the dissemination of AMR enterobacteria and microbiota modification of waterbodies^8^. Our findings indicate that the rural and urban water sources we examined in Brazil are widely contaminated with human feces and, according to CONAMA (ordinance n^o^ 274/00), these water sources are inappropriate for human consumption and recreational use. In rural areas, both human and animal feces may contribute to microbiota modification and, therefore, to the selection of resistant species^7^ ^,^ ^10^. In urban areas, high population density around waterbodies and lack of adequate sanitation influence the degree of contamination^4^ ^,^ ^11^. A previous study^1^ demonstrated that the human fecal content in CL was similar to that of raw sewage in Cleveland, Ohio, USA, using tracking of microbial source and DNA of human-indicative *Bacteroides* species. These findings require increased attention from local authorities and residents, as the waterbodies evaluated in this study serve as environmental reservoirs of AMR^12^ ^,^ ^8^.

We found AMR genes, even though there was no evidence of direct disposal of hospital waste in these waterbodies. The identified bacteria have an important association with human diseases (e.g., gastrointestinal colonization) and serve as a source of infection. The WHO has described them as a major public health concern, especially when associated with AMR^5^. In rural areas, we identified resistance genes, such as *bla*_*CTX-M,*_ *bla*_*OXA-48*_ , and *blaVIM*, using PCR; they may correlate with the spread of β-lactam-resistant bacteria in pig pens and via soil and domestic sewage. Our findings reinforce the presence of AMR in northeast Brazil, consistent with the results of studies in other rural environments^10^ ^,^ ^13^.

The urban settings in developing countries have high population density and are less efficient in sanitation^12^. Therefore, it was not surprising that we observed a higher AMR profile diversity in urban areas than in rural areas. Moreover, the presence of carbapenemase-producing and ESBL-positive bacteria is associated with worse prognosis of human infections. This type of resistance is frequently related to healthcare problems and many community-acquired infections^14^. Overall, in urban areas, our genotypic analysis revealed that the most frequent cephalosporinase gene was *bla*_*CTX-M*_ (22%) and carbapenemase gene was bla_*OXA-48*_ (33%). Studies have frequently reported these enzymes, probably owing to their easy dissemination, as they reside in mobile vectors, such as plasmids and transposable elements^15^.

Communal activities and poor infrastructure for sanitation pollute the surrounding aquatic environments. Therefore, rural and urban waterbodies are important reservoirs for the dissemination and selection of AMR enterobacteria and potential sites of acquiring severe and non-treatable human infections. Furthermore, poor sanitation aggravates this problem, especially in urban settings in developing communities. Consequently, more people are prone to become ill and infected with difficult-to-treat microorganisms. 
